# Application of bioabsorbable screw fixation for anterior cervical decompression and bone grafting

**DOI:** 10.6061/clinics/2016(06)06

**Published:** 2016-06

**Authors:** Bo Zhao, Xiaowen Qiu, Dong Wang, Haopeng Li, Xijing He

**Affiliations:** The Second Affiliated Hospital of Xi’an Jiaotong University, Department of Orthopedics, Xi’an/Shaanxi Province, PR China

**Keywords:** Bioabsorbable Screw, Cervical Spondylosis, Bone Grafting, Fixation

## Abstract

**OBJECTIVES:**

To examine the application of bioabsorbable screws for anterior cervical decompression and bone grafting fixation and to study their clinical effects in the treatment of cervical spondylosis.

**METHODS:**

From March 2007 to September 2012, 56 patients, 36 males and 20 females (38-79 years old, average 58.3±9.47 years), underwent a novel operation. Grafts were fixed by bioabsorbable screws (PLLA, 2.7 mm in diameter) after anterior decompression. The bioabsorbable screws were inserted from the midline of the graft bone to the bone surface of the upper and lower vertebrae at 45 degree angles. Patients were evaluated post-operatively to observe the improvement of symptoms and evaluate the fusion of the bone. The Japanese Orthopaedic Association (JOA) score was used to evaluate the recovery of neurological functions.

**RESULTS:**

All screws were successfully inserted, with no broken screws. The rate of symptom improvement was 87.5%. All of the grafts fused well with no extrusion. The average time for graft fusion was 3.8±0.55 months (range 3-5 months). Three-dimensional reconstruction of CT scans demonstrated that the grafts fused with adjacent vertebrae well and that the screws were absorbed as predicted. The MRI findings showed that the cerebrospinal fluid was unobstructed. No obvious complications appeared in any of the follow-up evaluations.

**CONCLUSIONS:**

Cervical spondylosis with one- or two-level involvement can be effectively treated by anterior decompression and bone grafting with bioabsorbable screw fixation. This operative method is safe and can avoid the complications induced by metal implants.

## INTRODUCTION

Anterior decompression and bone grafting with metal fixation is a standard operation, widely used for the treatment of cervical spondylosis. A number of device-related complications have been reported, such as screw malposition, extrusion, imaging artifacts and even plate abruption 1-3. According to *in vitro* biomechanical studies on bioabsorbable screws, absorbable screws can provide immediate stability. Compared to plate fixation, this new method exhibits higher stability in extension and comparable stability in flexion and axial rotation, but lower stability in lateral bending 4. We applied cross-interface fixation with bioabsorbable screws for the treatment of cervical spondylosis to avoid metal device-related complications. From March 2007 to September 2012, we performed this novel operation on 56 patients. Here, we present our surgical technique and clinical results.

## MATERIALS AND METHODS

### Ethics

Ethical board approval and consent from the study participants were obtained. This experiment was performed in accordance with the Helsinki Declaration of 1975, revised in 1983.

### Patients

The inclusion criteria for patients in this study were as follows:

1. a prolapse of the cervical intervertebral disc(s) involving a single segment or consisting of two segments ranging from C3-4 to C6-7;
2. significant neural symptoms, such as tingling or numbness that primarily affected the shoulders and arms or muscle weakness;
3. a reluctance to undergo metal device implantation;
4. an absence of other spinal diseases;
5. good general health.

Totally, 56 patients underwent this new type of operation. The average age of the 36 male and 20 female patients was 58.3±9.47 years (range 38-79 years). The patients included 21 cases of spinal cervical spondylotic myelopathy, 15 cases of cervical spondylotic radiculopathy and 20 cases of mixed types of cervical spondylosis. Single-level fusion was performed in 23 patients, including 10 at the C4-5 level and 13 at the C5-6 level. Fusion involving two levels was performed in 33 patients, including 18 at the C3-5 level, 10 at the C4-6 level and 5 at the C5-7 level. Pre-operative JOA scores ranged from 12 to 15, with 22 patients at 12 points, 18 at 14 points and 16 at 15 points (Table 1). Spinal cord compression (Figure 2E) and obvious neural symptoms such as numbness and/or muscle weakness were observed in all patients. All of the patients were well informed of the advantages and disadvantages of this new type of surgery.

### Surgical technique

**Figure 1:**
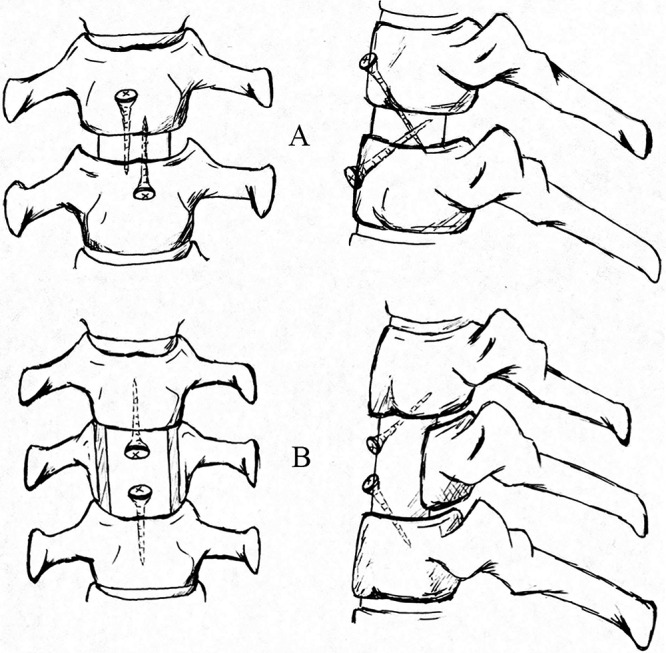
Schematics of the surgical technique performed at our institute using poly L/lactide screws. A: The directions of the screws in single-level fusion. B: The directions of the screws in two-level fusion.

**Figure 2:**
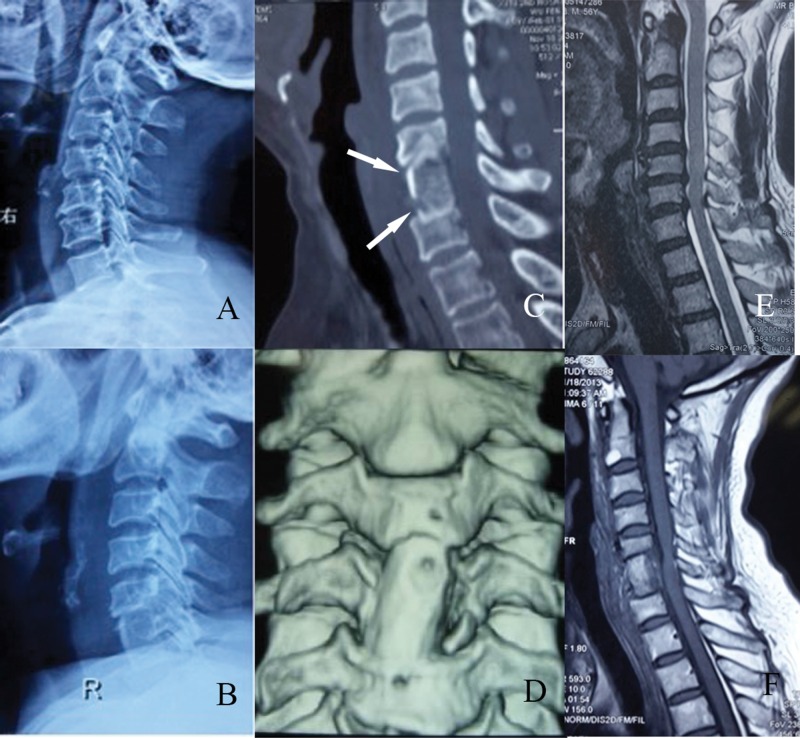
Imaging examinations of a patient who underwent C5-7 fusion. A: Radiograph, lateral view; pre-operation. B: Radiograph, lateral view; post-operation. C: Computed tomography scan, axial view; note the poly L/lactide screws (arrow). D: Three-dimensional reconstruction of the graft. E: Magnetic resonance imaging scan, axial view; pre-operation. F: Magnetic resonance imaging scan, axial view; post-operation.

After general anesthesia and intubation, patients were positioned with the neck slightly hyperextended. We accessed the target vertebrae and intervertebral disc using a standard Smith-Robinson approach with a 5 cm transverse incision 5. After the target vertebrae and disc were identified and located, we used curettes and laminectomy rongeurs to perform a single-level discectomy or a two-level subtotal vertebrectomy. The left and right sides of the vertebrae were retained at 5 mm during the subtotal vertebrectomy. The length of the deletion was determined by the decompression range (generally near the upper and lower endplates). We then measured the length of the intervertebral space or the lack of vertebrae and obtained an iliac strut graft with three cortical faces with the proper length and width. In cases involving two intervertebral spaces and a subtotal vertebrectomy, we drilled two holes that were 1 cm from each end and at an angle of 45 degrees to the surface of the graft, which allowed easier insertion and control of the direction of the screws. The graft was inserted into the groove of the vertebra with the cortex forward toward the outside. We continued drilling holes in the upper and lower vertebrae through the holes in the graft and inserted two fully threaded absorbable screws after threading (Figure 1B). The graft was well fixed and the screws did not penetrate the posterior cortex of the upper and lower vertebral bodies. In cases where a simple discectomy was performed, the height of the space after decompression was 1-1.5 cm. After the graft was placed into the space, two screws were inserted into the upper and lower vertebral body at 45 degree angles toward the graft (Figure 2A). All of the grafts were harvested from autologous iliac bone and the bioabsorbable screws were composed of poly L/lactide (PLLA) and fully threaded with a diameter of 2.7 mm (Bionx Implants Inc.). Finally, a drainage tube was placed and the incision was closed.

### Postoperative management

A neck collar was necessary to limit movement of the cervical spine after surgery. Two days after surgery, patients were allowed to be in a semi-sitting position and patients can walk on their own on the third day. A postoperative X-ray was obtained to observe the graft and fixation. The neck collar was removed 2-3 weeks after surgery. All patients were followed-up every month in our outpatient department to observe the improvement of symptoms and to evaluate the fusion of the bone graft. The JOA score was used to evaluate the recovery of neurological functions. A three-dimensional reconstruction of the CT scan was requested to evaluate the condition of the bioabsorbable screws and to determine whether the graft fused well with nearby vertebrae.

## RESULTS

All of the screws were inserted successfully and no screws were broken. The operation time ranged from 0.6 to 2.2 hours (average 1.2±0.37 hours). Average blood loss during the operation was 133±64 ml (range 50-350 ml). No complications occurred during surgery, such as hematoma, choking, esophageal injury, nerve injury, dural tearing, or cerebrospinal fluid leakage. All of the patients were followed-up for at least 24 months and no patient was lost. In the follow-up, no patients presented foreign body sensations, systemic adverse reactions, or graft prolapse or collapse. No complications such as screw breakage or esophageal injury were encountered. When the symptoms were evaluated by the Japanese Orthopaedic Association (JOA) score, the recovery rate (RR) of 39 patients was above 75%, the RR of 10 patients was 50%-74% and the RR of 7 patients was 25%-49%. RRs higher than 75% are excellent; RRs between 50% and 74% were considered good; and RRs between 25% and 49% were considered fair. The rate of excellent and good improvement was 87.5%. The average duration of graft fusion was 3.8±0.55 months (range 3-5 months, Table 2). X-rays and CT scans showed that the cervical curvature was normal and the height of the vertebrae exhibited no obvious loss (Figure 2B/C). Three-dimensional reconstructions of CT scans demonstrated that the graft fused well with adjacent vertebrae and that the screws were absorbed as predicted (Figure 2D). The MRI findings showed that the cerebrospinal fluid was unobstructed (Figure 2F). No obvious complications appeared throughout the follow-up evaluations.

## DISCUSSION

The bioabsorbable screws were composed of PLLA, which is created by polymerization with carbon, hydrogen and oxygen under certain conditions. The PLLA screws will swell upon exposure to water and are completely hydrolyzed into water and carbon dioxide *in vivo*. The type of screw used was 22 mm long with a diameter of 2.7 mm and a head diameter of 8 mm. The initial bend strength was 260 MPa 6,7; the modulus of elasticity was 5-14 GPa, approximately equal to that of the cancellous bone. Fourteen weeks after surgery, 88% of the bend strength was retained, which decreased to 70% after 50 weeks. The screws were completely absorbed over time, ranging from 32 weeks to 4 years 7. The degradation process is similar to the healing process of the cancellous bone fracture 8,9. In addition, the screws do not cause artifacts similar to metal plates.

Bioabsorbable material has good biocompatibility, is non-toxic and exhibits a low incidence of rejection 10-13. The idea of applying bioabsorbable screws to anterior cervical graft fixation was inspired by interference screw fixation. Kim et al. reported the use of bioabsorbable screws in cervical anterior fusion in 62 patients. In their method, the graft was inserted into the area of deletion resulting from decompression 14. The bioabsorbable screws were inserted into the four corners of the vertebral bodies and graft, perpendicular to the coronal plane. No screw or graft extrusions and no surgery-related infections were observed. The patients were able to walk a few hours after the operation and wore a simple neck collar for several days. In this study, the fixation method used was cross-interface fixation. Absorbable screws crossed the interfaces between the graft and the upper and lower vertebral endplate at an angle of 45 degrees to the coronal plane. In contrast to Kim’s method, this new method of fixation only requires two bioabsorbable screws. Zhang Jian et al. performed a biomechanical evaluation of this type of fixation compared to a traditional plate fixation in fresh human cadaveric cervical spines. They found that absorbable screws could provide immediate stability. Compared to plate fixation, this new method had higher stability in extension and comparable stability in flexion and axial rotation, but lower stability in lateral bending 4. In the present study, preliminary observations verified that this new method of fixation could provide the stability required for graft fusion in the treatment of cervical spondylosis. The 45-degree angle was considered easy for insertion while maintaining sufficient force to hold the graft. The optimal angle will be determined in subsequent examinations.

Anterior cervical decompression, grafting and plate fixation are commonly used in the treatment of cervical disease. However, most of the lock plate fixations are types of static fixations, compared to interface screw fixation. Because the modulus of elasticity of the metal screw is much greater than that of the cancellous bone, it is easy for the screws to cut the cancellous bone. This may cause the screws and even the entire fixation system to loosen, a common complication in long segment fixation, revealing a weakness of anterior cervical fixation with metal plates.

Compared to the anterior cervical plate technique, the most prominent advantage of the current technique is that absorbable screws are degraded as long as the bone graft fuses. This avoids metal device-related complications and reduces concerns about metal devices remaining in the body. Due to their biomechanical properties and *in vivo* swelling, these screws are ideal for cancellous bone fixation. Absorbable screws can avoid the stress shielding caused by metal devices because their modulus of elasticity is approximately equal to that of cancellous bone. Dynamic fixation is conducive to the growth of callus and fracture healing 9,15. Compared to the simple graft (no screws, Smith & Robbinson) 5, this fixation technique using bioabsorbable screws offers much more strength and costs little money. Use of this new type of fixation is a good balance between economic factors and the need for strength.

The longitudinal stress on the cervical spine is weak due to its mechanical characteristics; therefore, it can be stabilized when the proper graft is fully embedded after the distraction device is released 16. Fixation with bioabsorbable screws can meet the basic requirements for graft fusion. A neck collar is necessary to limit the movement of the cervical spine, especially during lateral bending, because fixation by bioabsorbable screws is weaker than anterior plate fixation with regard to lateral bending stress 4,17. Anterior cervical decompression and graft fixation will generally affect the curvature of the cervical spine. In this study, the follow-up time was not sufficient to evaluate the degeneration of adjacent segments and the effect on the reconstruction of cervical curvature. As the segment of decompression and fixation is lengthened, the stress that fixation materials undergo will significantly increase. Because this is a novel technique, patients whose procedure involved one or two spaces were selected and the fixation method was shown to be reliable. Biomechanical research and clinical observation of long segment fixation with absorbable screws will be undertaken.

No obvious adverse reactions of complications were encountered. Moreover, this type of fixation is less expensive for patients. This method of operation only requires two bioabsorbable screws instead of metal plates with several metal screws, which are very expensive. In addition, it can reduce patient concerns about metal devices remaining in the body. Thus, a secondary operation is not needed to remove the metal devices. The results of this study validated the application of cross-interface fixation with bioabsorbable screws for cervical spondylosis involving one or two intervertebral spaces. Possible future applications include single vertebral fracture, lower cervical dislocation, simple vertebral tumor, or vertebral tuberculosis.

Three factors should also be considered to optimize recovery rates. First, a neck collar should be worn for 2-3 weeks to avoid graft loosening because the lateral bending and rotational stress tolerance are weaker than in anterior plate fixation. However, wearing a neck collar for extended periods weakens the muscles of the neck. Second, this technique is a type of interface fixation. Therefore, an important measure for reducing the stress and avoiding screw breakage is to embed the graft exactly into the area of deletion resulting from decompression. Third, an autologous iliac strut needs to be harvested. Therefore, the incision used to collect the strut should be well cared for to prevent complications such as infection.

Cervical spondylosis with one- or two-level involvement can be effectively and economically treated by anterior decompression and bone grafting with bioabsorbable screw fixation. This operative method is safe and can avoid the complications induced by metal implants.

In this work, we evaluated the clinical effect of fixation by bioabsorbable screws after anterior decompression for the treatment of cervical spondylosis. This method of the fixation with bioabsorbable screws was used as a novel treatment for cervical spondylosis and the results showed that this new type of operation is safe and avoids metal device-related complications.

## AUTHOR CONTRIBUTIONS

Zhao B designed the study, acquired data, edited the manuscript and critically reviewed the final version of the manuscript. Qiu XW acquired data, conducted the statistical analysis, drafted the manuscript and critically reviewed the final version of the manuscript. Wang D, He XJ conceptualized the study, organized the data, edited the manuscript, and critically reviewed the final version of the manuscript. Li HP was the senior author and provided supervision in addition to the surgical treatment/technique and patient group selection. All of the authors read and approved the final manuscript.

## Figures and Tables

**Table 1:** General patient information.

	Number (%)
**Gender**	
Male	36 (64.8)
Female	20 (35.8)
Age (years)	Mean 58.3±9.47 (range 38-79)
**Type**	
Cervical spondylotic myelopathy	21 (37.5)
Cervical spondylotic radiculopathy	15 (26.7)
Mixed Type	20 (35.8)
Fusion level	
Single	23 (41.1)
Double	33 (58.9)
**Operative Segments**	
C4/5	10 (17.9)
C5/6	13 (23.2)
C3-5	18 (32.1)
C4-6	10 (17.9)
C5-7	5 (8.9)
**JOA Score**	
12 points	22 (39.3)
14 points	18 (32.1)
15 points	16 (28.6)

**Table 2:** Results.

**Time of operation (hours)**	Mean 1.2±0.37 (range 0.6-2.2)
**Blood loss (ml)**	Mean 133±64 (range 50-350)
**Recovery Rate (RR) by JOA**	Number of patients (%)
Excellent (≥75%)	39 (69.6)
Good (50-74%)	10 (17.9)
Fair (25-49%)	7 (12.5)
